# High sensitivity C-reactive protein and cerebral white matter hyperintensities on magnetic resonance imaging in migraine patients

**DOI:** 10.1186/1129-2377-16-9

**Published:** 2015-01-16

**Authors:** Aynur Yilmaz Avci, Hatice Lakadamyali, Serap Arikan, Ulku Sibel Benli, Munire Kilinc

**Affiliations:** Department of Neurology, Baskent University, Saray Mah, Yunusemre cad, No. 1, Alanya-Antalya, 07400 Ankara Turkey; Department of Radiology, Baskent University, Ankara, Turkey; Department of Biochemistry, Baskent University, Ankara, Turkey

**Keywords:** Headache, Inflammation, Vascular disease, Pathophysiology

## Abstract

**Background:**

Migraine is a common headache disorder that may be associated with vascular disease and cerebral white matter hyperintensities (WMHs) on magnetic resonance imaging (MRI) scan. High sensitivity C-reactive protein (hs-CRP) is a marker of inflammation that may predict subclinical atherosclerosis. However, the relation between migraine, vascular risks, and WMHs is unknown. We evaluated hs-CRP levels and the relation between hs-CRP level and WMHs in adult migraine patients.

**Methods:**

This case–control study included 432 subjects (216 migraine patients [without aura, 143 patients; with aura, 73 patients]; 216 healthy control subjects without migraine; age range 18–50 y). Migraine diagnosis was determined according to the International Classification of Headache Disorders II diagnostic criteria. The migraine patients and control subjects had no known vascular risk factors, inflammatory disease, or comorbid disease. The presence and number of WMHs on MRI scans were determined, and serum hs-CRP levels were measured by latex-enhanced immunoturbidimetry.

**Results:**

Mean hs-CRP level was significantly greater in migraine patients (1.94 ± 2.03 mg/L) than control subjects (0.82 ± 0.58 mg/L; *P* ≤ .0001). The mean number of WMHs per subject and the presence of WMHs was significantly greater in migraine patients (69 patients [31.9%]; 1.68 ± 3.12 mg/dL) than control subjects (21 subjects [9.7%]; 0.3 ± 1.3; *P* ≤ .001). However, there was no correlation between hs-CRP level and WMHs in migraine patients (r = 0.024; not significant). The presence of WMHs was increased 4.35-fold in migraine patients (odds ratio 4.35, *P* ≤ .001).

**Conclusions:**

High hs-CRP level may be a marker of the proinflammatory state in migraine patients. However, the absence of correlation between hs-CRP level and WMHs suggests that hs-CRP is not causally involved in the pathogenesis of WMHs in migraine patients. The WMHs were located mostly in the frontal lobe and subcortical area.

## Background

Migraine is a common neurologic disorder, typically characterized by recurrent attacks of debilitating headache and symptoms of autonomic nervous system dysfunction. In one third patients, migraine attacks are accompanied by transient focal neurologic aura symptoms. The frequency of migraine is 3-fold greater in women than men [1]. Migraine prevalence is 8.6% in males, 17.5% in females, and 13.2% overall in the United States [2]. In Turkey, migraine prevalence is 8.5% in males and 24.6% in females, and the 1-year prevalence of migraine (16.4%) is similar or higher than the prevalence worldwide [3]. The risk of having migraine is greater in women aged < 45 years, and hormonal effects may be a causal factor for this female predominance [4]. Migraine is associated with an increased risk of developing cardiovascular disease and a 2-fold increased risk of developing ischemic stroke [4–7]. The relation between migraine and the risk of vascular disease may be explained, in part, by the higher prevalence of multiple risk factors in migraine patients [4–6]. Additionally, the association between migraine with aura and ischemic stroke is more apparent for individuals without vascular risk factors [8–10]. Migraine also has also been associated with hemorrhagic stroke [11].

Migraine is a neurovascular disorder associated with cortical spreading depression, neurogenic inflammation, and cranial vascular contractile dysfunction. Activation of brain tissue causes release of peptides from the perivascular trigeminal regions that cause inflammation and dilation of extraparenchymal vessels. Repeated migraine attacks are associated with inflammatory arteriopathy of the cranial vessels [12, 13]. In migraine, specific abnormalities of inflammatory marker levels in the systemic circulation have been observed, including increased levels of C-reactive protein (CRP), interleukins, and adhesion molecules [13–15].

Migraine is a risk factor for white matter hyperintensities (WMHs), which are infarct-like lesions associated with volume changes in the brain (grey matter and white matter regions) [16]. Incidental findings on brain magnetic resonance imaging (MRI) scans are common, and incidental findings detection is more likely using high resolution MRI sequences than standard resolution sequences [17–20]. The pathophysiology of WMHs is unknown. Increased age and atherosclerosis may be the main risk factors for the development of WMHs [17, 20, 21]. In migraine, the duration of disease and attack frequency are important in the development of WMHs, and comorbid disease also may contribute to the development of WMHs [18, 20, 21]. In migraine, cumulative effects of repeated intracerebral hemodynamic changes may contribute to the development of WMHs, including oligemia, focal hypoperfusion with ischemia, and hypoxia below the ischemic threshold [22].

High sensitivity C-reactive protein (hs-CRP) is a marker of inflammation, and hs-CRP levels may increase in vascular diseases such as myocardial infarction and ischemic stroke or in healthy individuals who have no cardiovascular disease [23]. The hs-CRP level has prognostic use in the Framingham coronary risk score, severity of metabolic syndrome, severity of hypertension, and patients who have or do not have subclinical atherosclerosis [23, 24]. The association of CRP level with migraine has been shown in small case–control studies of migraine with vascular risk factors and a large prospective cohort study of women aged > 45 years [15, 25–27]. Higher CRP levels were associated with the presence and progression of periventricular and subcortical WMHs, independent of cardiovascular risk factors and carotid atherosclerosis, in old nondemented patients [28–30].

The complex mechanisms involved in migraine and mechanisms linking migraine and vascular risks are incompletely understood [4, 5]. The distribution of brain WMHs in patients who have migraine is unclear, and limited information is available about the complex relation between migraine, ischemia, and WMHs [18–20].

We hypothesized that hs-CRP levels may be associated with migraine disease, and that high hs-CRP levels also may be associated with the presence and distribution of cerebral WMHs on brain MRI scans in migraine patients. The purpose of the present study was to analyze the relation between hs-CRP levels and WMHs in migraine patients.

## Methods

### Subjects

This case–control clinical study was performed with patients who had migraine newly diagnosed at the neurology outpatient clinics of Baskent Medical Faculty from October 2011 to December 2013 and who had brain MRI scans as a part of their evaluation. Inclusion criteria were duration of migraine symptoms ≥ 1 year, headache frequency ≥ 2 attacks/month, and absence of any known vascular risk factors, inflammatory disease, chronic illness, metabolic disease, or infections. Patients were excluded for (1) migraine duration < 1 year; (2) history of cerebrovascular or cardiovascular disease, arterial hypertension (blood pressure > 130/80 mm Hg), diabetes mellitus, or hyperlipidemia (low-density lipoprotein cholesterol ≥ 160 mg/dL); (3) body mass index < 18 kg/m^2^ or > 35 kg/m^2^; (4) abnormal plasma hs-CRP level (>10 mg/L); (5) smoking cigarettes > 1 pack/day; (6) current pregnancy, lactation, or hormonal contraceptive use; (7) alcohol or substance abuse; (8) drug use such as antiplatelet agents, anticoagulants, statins, or hormonal drugs; (9) renal, metabolic, psychiatric, inflammatory, infectious, or immune disease; (10) musculoskeletal disorders or fibromyalgia; or (11) possible “symptomatic migraine” in which the MRI showed arteriovenous malformations, ischemic infarcts, brain tumors, or other conditions that may be associated with migraine. In addition, patients who had high levels of hs-CRP (≥10 mg/L) were excluded from the study because high levels of hs-CRP (>10 mg/L) may represent nonspecific inflammation and lack positive predictive value [23, 24]. There were 300 consecutive migraine patients considered for the study, and 84 patients were excluded (infection, 17 patients; thyroid disease, 12 patients, thyroid stimulating hormone, range 3.5 - 4.9 μIU/mL; no laboratory tests available, 11 patients; declined MRI scan, 10 patients; low-density lipoprotein cholesterol > 160 mg/dL, 9 patients; positive antinuclear antibody, 9 patients; hs-CRP > 10 mg/L, 8 patients; antidouble-stranded DNA autoantibody, 5 patients; silent lacunar infarct, 3 patients). The other 216 consecutive, newly diagnosed migraine patients were included in the study (165 women [76%] and 51 men [24%]; age range, 18–50 y [mean age, 31 ± 7 y]).

Healthy control subjects (216 subjects: 150 women [69.4%] and 66 men [30.6%]) aged between 18 and 50 years (mean age, 32.46 ± 7.54) were recruited consecutively from hospital staff, laboratory staff, relatives of patients, and the general population. Inclusion criteria for the control subjects were (1) absence of headaches such as migraine, tension-type headache, or cluster headache; (2) absence of other neurologic or systemic disease; and (3) presence of a match with migraine patients by age (±2 y), sex, body mass index, education level, and smoking habits. Exclusion criteria for control subjects were the same as for the migraine group. The study was approved by the local ethics committee of the Medical Faculty of Baskent University Hospital, and all migraine and control subjects gave informed consent to participate in the study and have MRI scans and laboratory tests.

### Evaluation

Patients were diagnosed as having migraine according to the criteria of the International Classification of Headache Disorders II [1]. A detailed history of migraine was obtained including disease duration (y), age at onset, average duration of current headache (h), presence of aura, trigger factors, accompanying symptoms, frequency per month, and location and severity of pain. Severity of headache was evaluated with visual analog score (range, 1 [minimum pain] to 10 [maximum pain]). Migraine headache attack frequency was defined as the number of attacks per month. All patients and control subjects received a complete physical and neurologic examination. Comorbidities (coronary artery disease, stroke, diabetes mellitus, or thyroid disease) and intercurrent illnesses such as respiratory or urinary infections were determined from the patient history, physical examination, and laboratory tests (biochemical and hematologic tests). Blood pressure, body weight, height, smoking habits, and education level were recorded for all migraine patients and control subjects. No migraine patients took any medication within 3 days before blood sampling. Patients previously had used medications for acute pain such as acetaminophen, nonsteroidal anti-inflammatory drugs, triptans, or caffeine for headache, but patients who used analgesics daily were excluded from the study. Patients who were treated for migraine prophylaxis with drugs such as propranolol, topiramate, or valproic acid were excluded.

### White matter hyperintensities

All 432 participants had cerebral MRI brain scanning (1.5 Tesla, Siemens Magnetom Vision Plus, Siemens, Munich, Germany) with the orbitomeatal line as reference. The scans included ≥ 3 sequences: sagittal T1-weighted, axial T2-weighted, and axial fluid attenuated inversion recovery (FLAIR) images. The slice thickness was 5 mm, the gap was 1 mm, and no intravenous contrast was used.

All MRI scans were reviewed and scored by a radiologist who was blinded to the clinical details. The scans were visually assessed for the presence and features of WMHs including appearance, number, size, distribution (infratentorial or supratentorial), and anatomic location. The number and size of the WMHs were determined on the FLAIR images and grouped according to location and distribution. Subgroups were delineated according to the distribution of the WMH following the methodology previously described in multiple sclerosis patients [31]. These subgroups were juxtacortical, subcortical, and periventricular. The locations of WMHs were defined as frontal, parietal, temporal, occipital, or infratentorial. Periventricular WMHs were defined as being anterior, posterior, or located at the lateral band. We included focal and punctate hyperintensities (size < 9 mm). Confluent and large hyperintensity lesions (>9 mm) were excluded. There were no WMHs at the corpus callosum. The McDonald and Barkhof MRI diagnostic criteria for dissemination in space in multiple sclerosis were applied to each patient to determine whether the criteria were satisfied [31, 32].

Migraine and control subjects who had WMHs that were detected on brain MRI were evaluated with laboratory tests for vasculitis (anticardiolipin antibodies, antinuclear antibody, lupus anticoagulant, antidouble-stranded DNA autoantibody, and C3 and C4 levels). Migraine and control subjects who had WMHs underwent cardiac examination and transthoracic echocardiography to exclude patent foramen ovale and atrial septal defect; only 1 patient was excluded because of atrial septal defect on echocardiography.

### High sensitivity C-reactive protein

Blood samples were obtained from the antecubital vein from control subjects and during a headache-free period from migraine patients. To exclude the potential effects of a recent attack, migraine patients had been free of migraine attack for ≥ 3 days before blood sampling. No subjects had taken anti-inflammatory drugs for ≥ 3 days before the study because these drugs may be associated with improved endothelial function and might have affected the results. Phlebotomy tubes contained no anticoagulant. Blood was centrifuged at 3000 × *g* for 10 minutes and stored at -20°C until analysis. Serum hs-CRP was measured by latex-enhanced immunoturbidimetry using monoclonal anti-CRP antibodies (Architect C 800, Abbott Diagnostic Systems, Abbott Park, IL, USA) (hs-CRP reference level, ≤ 5 mg/L).

### Statistical analysis

Data analysis was performed with statistical software (IBM SPSS Statistics for Windows, Version 21.0, IBM Corp., Armonk, NY, USA). Statistical analysis of the numeric parameters that were normally distributed was performed with independent *t* test. Data that did not satisfy normal distribution approximation were analyzed with nonparametric Mann–Whitney test. Average values were reported as mean ± standard deviation (SD), and statistical analysis was performed with median values. Categorical and ordinal data were analyzed with Pearson chi-square and Fisher exact chi-square tests. Correlation analysis was performed with Spearman rank correlation. Factors affecting hs-CRP were investigated with regression analysis. However, to prove hypotheses of the regression analysis, transformation to the hs-CRP variable was applied to satisfy the normal distribution condition of the parameter. Factors affecting the presence of WMHs were analyzed with multiple logistic regression. In addition, descriptive statistics for categorical variables were specified as number (%), and median statistics for numeric variables were reported with range (minimum to maximum) and mean ± SD. In all analyses, statistical significance was defined by *P* ≤ .05 and decisions were at the 95% confidence level.

## Results

In the 216 consecutive, newly diagnosed migraine patients who were included in the study, migraine without aura was diagnosed in 143 patients (66%) and migraine with aura was diagnosed in 73 patients (34%) (Table 1). The migraine and control groups were similar in mean age, body mass index, education, and frequency of smokers (Table 1). Frequency of family history of migraine and mean hs-CRP levels were similar between migraine patients with or without aura and were greater in migraine patients than control subjects (Table 1). Mean duration of migraine disease was statistically significantly greater in patients who had migraine with than without aura (Table 1). There was no statistically significant difference between the migraine groups in headache localization, headache duration, visual analog score, headache frequency, medication, education level, or smoking habits (Table 1).

**Table 1:** Characteristics of participants in study of migraine*

Characteristic		Control (n = 216)	Migraine without aura (n = 143)	Migraine with aura (n = 73)	***P*** ≤^†^
Age (y)		32 ± 4.6	30.9 ± 7.6	32.6 ± 6.7	NS
Body mass index (kg/m^2^)		24.3 ± 3.3	24.4 ± 3.9	25 ± 4.1	NS
Sex					.002^‡^
Female		150 (69.4)	105 (73.4)	60 (82.2)	
Male		66 (30.6)	38 (26.6)	13 (17.8)	
hs-CRP (mg/L)	Mean ± SD	0.82 ± 0.58	1.76 ± 1.86	2.31 ± 2.30	.001^‡^
	Median (min-max)	0.69 (0.10-2.90)	1.09 (0.10-9.51)	1.48 (0.10-9.92)	
hs-CRP (no. of subjects [%])	<1 mg/L	147 (68.1)	65 (45.5)	28 (38.4)	.001^‡^
	1-3 mg/L	69 (31.9)	53 (37.1)	25 (34.2)	
	>3 mg/L	0 (0)	25 (17.5)	20 (27.4)	
WMHs present (no. of subjects [%])		21 (9.7)	44 (30.8)	25 (34.2)	.001^‡^
No. of WMHs per subject	Mean ± SD	0.34 ± 1.27	1.62 ± 3.07	1.66 ± 3	.001^‡^
	Median (min-max)	0 (0–10)	0 (0–16)	0 (0–15)	
No. of subjects with WMHs	0 WMH	195 (90.3)	99 (69.2)	48 (65.8)	.001^‡^
	1-2 WMHs	8 (3.7)	9 (6.3)	4 (6.5)	
	≥3 WMHs	13 (6.0)	35 (24.5)	21 (28.8)	
Migraine disease duration (y)	Mean ± SD	-	6.3 ± 5.2	10.1 ± 7	.001^§^
	Median (min-max)	-	5 (1 – 30)	10 (1 – 30)	
Headache localization	Half head	-	47 (32.9)	20 (27.4)	NS
	Entire head	-	96 (67.1)	53 (72.6)	
Visual analog score	Mean ± SD	-	7.3 ± 1.8	7.8 ± 1.8	NS
	Median (min-max)	-	7 (3–10)	8 (4–10)	
Headache frequency (no./mo)	Mean ± SD	-	7.6 ± 5.6	7.7 ± 5.6	NS
	Median (min- max)	-	6 (2–20)	5 (2–20)	
Headache duration (h)	Mean ± SD	-	25,46 ± 27,69	26,11 ± 27,62	NS
	Median (min - max)	-	18.0 (1–168)	18.0 (1–120)	
Medication	Acetaminophen	-	41 (28.7)	22 (30.1)	NS
	Nonsteroidal anti-inflammatory drug	-	86 (60.1)	41 (56.2)	
	Ergotamine	-	7 (4.9	8 (11)	
	Triptan	-	9 (6.3)	2 (2.7)	
Education completed	Primary school	21 (25.6)	41 (28.9)	24 (32.9)	NS
	Secondary school	6 (7.3)	15 (10.6)	8 (11)	
	High school	29 (35.4)	41 (28.9)	22 (30.1)	
	University	26 (31.7)	45 (31.7)	19 (26)	
Family history of headache		23 (28)	110 (76.9)	58 (79.5)	.001^‡^
Smoking (≤1 pack/d)		20 (24.4)	35 (24.5)	18 (24.7)	NS
Thyroid stimulating hormone (μIU/mL)	Median ± SD	1.43 ± 0.73	1.42 ± 0.71	1.47 ± 0.77	NS
	Median (min - max)	1.26 (0.37-4.03)	1.25 (0.35-3.90)	1.41 (0.44-4.25)	
Ejection Fraction (%)	Median ± SD	65.67 ± 4.76	65.24 ± 3.61	64.44 ± 4.33	NS
	Median (min - max)	65 (60–75)	65 (60–74)	65 (60–75)	

*Data reported as mean ± SD, number of subjects (%), or median (range, minimum to maximum). *Abbreviations:* *hs-CRP* high sensitivity C-reactive protein, *WMH* white matter hyperintensity.

^†^NS, not significant (*P* > .05).

^‡^Significant difference between migraine patients and control subjects.

^§^Significant difference between migraine patients without or with aura.

The presence of WMHs and mean number of WMHs per subject were significantly higher in migraine patients (69 patients [31.9%]; 1.68 ± 3.12 mg/dL) than control subjects (21 subjects [9.7%]; 0.3 ± 1.3; *P* ≤ .0001) (Table 1). In the 69 migraine patients who had WMHs, 67 patients (97%) had supratentorial WMHs and 2 patients (3%) had infratentorial WMHs. In 15 of 69 (10.4%) migraine patients who had WMHs, the WMHs were present in > 1 anatomic location. In migraine patients, WMH diameter was ≤ 3 mm in 63 patients (91%) and 4 to 9 mm in 6 patients (9%). In all control subjects who had WMHs (21 patients [9.7%]), WMH diameter was ≤ 3 mm, and no infratentorial lesions were present.

The distribution of WMHs was significantly different between migraine and control subjects (Table 2). The presence and the number of WMHs per subject (juxtacortical, subcortical, and periventricular) were significantly higher in migraine patients than control subjects (*P* ≤ .001) (Table 2). The WMHs in the migraine and control groups were detected most frequently in the frontal lobe and least frequently in the occipital lobe (Table 2) (*P* ≤ .05). In the control group, the WMHs were detected only in the subcortical region (Table 2). Frequencies of juxtacortical and subcortical WMHs were similar between migraine patients with and without aura (not significant). Only migraine patients with aura had periventricular WMHs (Table 2), which were located in the anterior horn of the lateral ventricles; no patients had WMHs in the lateral ventricular bands or posterior horn of lateral ventricles (Table 2). In the 69 migraine patients, the WMHs were contiguous with the cortex in 18 patients (26%) and with the periventricular structure in 3 patients (4%) (Table 2). In 69 migraine patients who had WMHs, only 1 patient satisfied the 2010 McDonald criteria [32]. No patients satisfied the Barkhof criteria or radiologically isolated syndrome criteria [31, 33].

**Table 2:** Anatomic location and distribution of juxtacortical, subcortical, and periventricular white matter hyperintensities in migraine and control subjects*

White matter hyperintensities		Control (n = 216)		Migraine without aura (n = 143)		Migraine with aura (n = 73)		***P*** ≤^†^
Juxtacortical WMHs		0 (0)		12 (8.4)		6 (8.2)		.0001^‡^
Juxtacortical WMHs	Frontal	0 (0)		7 (4.9)		3 (4.1)		.0001^‡^
	Parietal	0 (0)		4 (2.8)		2 (2.7)		.03^‡^
	Temporal	0 (0)		1 (0.7)		1 (1.4)		NS
	Occipital	0 (0)		0 (0.5)		0 (0.5)		-
Juxtacortical WMHs		0.0 ± 0.0	0 (0–0)	0.1 ± 0.48	0 (0–3)	0.14 ± 0.53	0 (0–3)	0.0001^‡^
Juxtacortical WMHs	Frontal	0.0 ± 0.0	0 (0–0)	0.09 ± 0.41	0 (0–3)	0.05 ± 0.28	0 (0–2)	0.0001^‡^
	Parietal	0.0 ± 0.0	0 (0–0)	0.04 ± 0.26	0 (0–2)	0.07 ± 0.42	0 (0–3)	.02^‡^
	Temporal	0.0 ± 0.0	0 (0–0)	0.01 ± 0.08	0 (0–1)	0.01 ± 0.12	0 (0–1)	NS
	Occipital	0.0 ± 0.0	0 (0–0)	0.0 ± 0.0	0 (0–0)	0.0 ± 0.0	0 (0–0)	-
Subcortical WMHs		21 (9.7)		41 (28.7		24 (32.9)		.0001^‡^
Subcortical WMHs	Frontal	20 (9.3)		36 (25.2)		22 (30.1)		.0001^‡^
	Parietal	6 (2.8)		24 (16.8)		13 (17.8)		.0001^‡^
	Temporal	0 (0)		2 (1.4)		4 (5.5)		.03^‡^
	Occipital	0 (0)		1 (0.7)		0 (0)		-
Subcortical WMHs		0.34 ± 1.28	0 (0–10)	1.46 ± 2.9	0 (0–14)	1.60 ± 2.99	0 (0–13)	0.0001^‡^
Subcortical WMHs	Frontal	0.31 ± 1.19	0 (0–10)	0.94 ± 1.99	0 (0–11)	1.10 ± 2.12	0 (0–11)	.0001^‡^
	Parietal	0.03 ± 0.17	0 (0–1)	0.47 ± 1.32	0 (0–8)	0.42 ± 1.19	0 (0–8)	.0001^‡^
	Temporal	0.0 ± 0.0	0 (0–0)	0.08 ± 0.36	0 (0–2)	0.08 ± 0.36	0 (0–2)	0.014^‡^
	Occipital	0.0 ± 0.0	0 (0–0)	0.01 ± 0.17	0 (0–2)	0.0 ± 0.0	0 (0–0)	NS
Periventricular WMHs		0 (0)		0 (0)		3 (4.1)		.04^ǁ^
Periventricular WMH	Anterior	0 (0)		0 (0)		3 (4.1)		.04^ǁ^
	Posterior	0 (0)		0 (0)		0 (0)		-
	Lateral	0 (0)		0 (0)		0 (0)		-
Periventricular WMHs		0.0 ± 0.0	0 (0–0)	0.0 ± 0.0	0 (0–0)	0.04 ± 0.20	0 (0–1)	.02^ǁ^
Periventricular WMHs	Anterior	0.0 ± 0.0	0 (0–0)	0.0 ± 0.0	0 (0–0)	0.04 ± 0.20	0 (0–1)	.02^ǁ^
	Posterior	0.0 ± 0.0	0 (0–0)	0.0 ± 0.0	0 (0–0)	0.0 ± 0.0	0 (0–0)	-
	Lateral	0.0 ± 0.0	0 (0–0)	0.0 ± 0.0	0 (0–0)	0.0 ± 0.0	0 (0–0)	-

*Data reported as number of patients (%), mean ± SD, or median (range, minimum to maximum). *Abbreviation:* *WMHs* deep white matter hyperintensities.

^†^NS, not significant (*P* > .05).

^‡^Comparison between control subjects and migraine patients (with and without aura).

^ǁ^Comparison between migraine patients with and without aura.

There was no statistically significant correlation between hs-CRP level and WMHs in migraine and control subjects (r = 0.155; *P* > .001) (Table 3). In migraine patients, the hs-CRP levels were not significantly different between juxtacortical, subcortical, and periventricular WMHs (Table 4). There was no correlation between hs-CRP and headache characteristics (r < 0.042; not significant) (Table 4). The hs-CRP level was similar for females and males, and the presence and number of WMHs/subject were similar between females and males (Table 4). The WMHs (juxtacortical, subcortical, and periventricular) were not correlated with headache characteristics (migraine duration, visual analog score, or headache duration) (not significant).

**Table 3:** Relation between high sensitivity C-reactive protein and cerebral white matter hyperintensities on magnetic resonance imaging in migraine patients and control subjects*

Parameter		All participants (n = 432)		Control subjects (n = 216)		Migraine patients (n = 216)	
		r	***P*** ≤ ^†^	r	***P*** ≤ ^†^	R	***P*** ≤^†^
WMHs		0.155	.001	0.165	0.015	0.024	NS
Juxtacortical WMHs		0.062	NS	-	-	-0.003	NS
Juxtacortical WMHs	Frontal	0.101	0.036	-	-	0.077	NS
	Parietal	-0.003	NS	-	-	-0.068	NS
	Temporal	-0.007	NS	-	-	-0.037	NS
Subcortical WMHs		0.148	0.002	0.165	.015	0.018	NS
Subcortical WMHs	Frontal	0.132	0.006	0.145	.033	0.008	NS
	Parietal	0.110	0.022	0.095	NS	0.031	NS
	Temporal	0.075	NS	-	-	0.041	NS
	Occipital	0.049	NS	-	-	0.036	NS
Periventricular WMHs		0.074	NS	-	-	0.054	NS
Periventricular WMHs Anterior		0.074	NS	-	-	0.054	NS

*Data reported as correlation coefficient r between hs-CRP and parameter. *Abbreviation:* *WMHs* white matter hyperintensities.

^†^NS, not significant (*P* > .05).

**Table 4:** Relation between high sensitivity C-reactive protein and location of cerebral white matter hyperintensities, headache characteristics, and sex differences in migraine patients*

Parameters		hs-CRP (mg/L)		***P*** ≤^†^
Juxtacortical WMHs	Present	1.95 ± 2,22	1.13 (0.21 – 9.40)	NS
	Absent	1.94 ± 2,01	1.25 (0.10 – 9.92)	
Subcortical WMHs	Present	2.01 ± 2.20	1.35 (0.10 – 9.92)	NS
	Absent	1.91 ± 1,95	1.18 (0.19 – 9.80)	
Periventricular WMHs	Present	1.91 ± 0.83	2.04 (1.02 – 2.67)	NS
	Absent	1.94 ± 2.04	1.24 (0.10 – 9.92)	
Sex	Female	1.99 ± 2.14	1.21 (0.01 – 9.92)	NS
	Male	1.80 ± 1.62	1.35 (0.10 – 8.30)	
Migraine disease duration (y)	≤1	1.46 ± 2.04	0.75 (0.15 – 9.51)	NS
	2-6	1.92 ± 1.87	1.35 (0.10 – 9.40)	
	≥7	2.10 ± 2.18	1.31 (0.10 – 9.92)	
Headache duration (h)		r = -0.002		NS
Visual analog score		r = -0.028		NS
Headache frequency (no./mo)		r = 0.042		NS

*Data reported as mean ± SD, number of subjects (%), median (range, minimum to maximum), or correlation coefficient r between hs-CRP and parameters. *Abbreviations:* *hs-CRP* high sensitivity C-reactive protein, *WMH* white matter hyperintensity.

^†^NS, not significant (*P* > .05).

Migraine disease was associated with a 4.35-fold increased risk of presence of WMHs (Table 5). In addition, age 1 year was associated with a 1.06-fold increased risk of the presence of the WMHs (Table 5).

**Table 5:** Factors affecting the presence of white matter hyperintensity*

Parameter	Odds ratio (95% confidence interval)	***P*** ≤^†^
Migraine	4.35 (1.904 - 0.945)	.001^‡^
hs-CRP	1.05 (0.906 - 1.213)	NS
Age	1.06 (1.018 - 1.102)	.004^ǁ^
Body mass index	1.02 (0.942 - 1.100)	.NS

*Data reported as odds ration and 95% confidence interval. *Abbreviation:* *hs-CRP* high sensitivity C-reactive protein.

^†^NS, not significant (*P* > .05).

^‡^Migraine increased the risk of the presence of WMH by 4.35-fold.

^ǁ^Age 1 y increased the risk of the presence of WMH by 1.06-fold.

## Discussion

In the present study, serum hs-CRP levels were significantly higher in migraine patients than control subjects. Based on published Framingham coronary risk criteria, 57.9% migraine patients had moderate to high vascular risk [23, 24]. The present data did not show associations between hs-CRP level and distribution of WMHs in migraine patients (Table 3). The prevalence of WMHs was 4.35-fold higher in migraine patients than control subjects (Table 5). The WMHs primarily involved the subcortical region and frontal lobe (Table 2). Only 1 migraine patient satisfied the revised 2010 McDonald criteria for multiple sclerosis [32].

A unique feature of the present study design was the simultaneous assessment of the associations of hs-CRP and WMHs in migraine patients. The participants had no known vascular risk factors or inflammatory disease. We included patients aged < 50 years to minimize the effects of age-dependent WMHs. Limitations of the present study included the limited number of participants. In addition, an MRI scanner with a higher magnetic field than the scanner used may be more sensitive in detecting WMHs on FLAIR images. A more sensitive imaging protocol with thin slices (thickness, 3 mm) might have improved the detection of smaller lesions. Furthermore, some risk factors such as smoking and obesity were not strictly excluded; although we excluded heavy smokers (>1 pack/d), we did not exclude all cigarette smokers. There were no statistically significant differences between the migraine and control groups in smoking habits. Although all participants had body mass index ≤ 35 kg/m^2^, some participants had body mass index > 30 kg/m^2^, and CRP level may increase with obesity [34]. Nevertheless, we observed no significant differences between the migraine and control groups in body mass index. In addition, it may be important to study other markers of endothelial dysfunction or inflammation such as tissue plasminogen activator antigen, von Willebrand factor activity, homocysteine level, or inflammatory cytokine levels to further evaluate the association of hs-CRP in the development of WMHs in migraine patients. We did not perform carotid Doppler studies to evaluate the possibility of microemboli.

The observed high hs-CRP levels in migraine patients were consistent with previous reports (Table 1) [15, 25–27]. In contrast with the present study, the Reykjavik study reported that CRP levels were similar in migraine patients and control subjects; vascular risk factors and concomitant disease were not eliminated, and the mean age of participants was 55 years [35]. Furthermore, another study reported no association between hs-CRP levels and migraine, and patients had no vascular risk factors; however, the sample was small, mean age of participants was older than in the present study, and headache frequency and migraine disease duration were not reported [36]. In the present study, inclusion criteria ensured that headache frequency was ≥ 2 headaches/mo and migraine disease duration was > 1 year. Elevated hs-CRP level is a sensitive marker of inflammation and arteriosclerosis [23]. The higher mean level of hs-CRP in migraine patients than control subjects is evidence that inflammation may contribute to migraine (Table 1). In migraine, elevation of CRP may be caused by oxidative stress, leukocyte activation, and inflammatory dilation of blood vessels, and inflammatory cytokines are increased during acute attacks of migraine and between attacks [13, 14].

Although migraine is a risk factor for WMHs, incidental findings on brain MRI scans are common, prevalence increases with age, and detection is more likely using high resolution MRI sequences than standard resolution sequences [16, 17]. The prevalence of WMHs in adult migraine patients varies from 14% to 59% [16, 20, 21]. In the present study, WMHs were detected with 4.35-fold greater frequency on brain MRI scans in migraine patients than control subjects (Table 5). The reason for the association between headache and WMHs is unknown. The WMHs may be an indirect marker of focal cerebral hypoperfusion induced by migraine attacks. Repeated and prolonged oligemia during and after migraine attacks may affect the vulnerable small deep penetrating arteries, and the WMHs may represent minor brain injury caused by reduced local perfusion [22, 37].

In the present study, hs-CRP levels were not associated with the presence and severity of WMHs on brain MRI scans in migraine patients (Tables 2 and 5), consistent with previous studies [30, 38, 39]. In contrast with our study, several previous studies reported a relation between hs-CRP levels and WMHs in elderly nondemented patients with unknown migraine history [28, 29]. The Cardiovascular Health Study reported that CRP levels were associated with the presence of WMHs; participants were aged > 65 years, vascular risk factors were not eliminated, and patients had cerebral ischemic infarcts [29]. The Rotterdam Scan Study described a relation between CRP and WMHs (severity and 3-year progression of WMHs); the participants were aged 60 to 90 years and had cardiovascular risk factors [28]. Increased age and vascular risk factors have been associated with high levels of hs-CRP and the presence of WMHs [21, 23, 24]. Differences in the composition of study participants may have been important. In the present study, the participants were younger and apparently healthier than those of previous studies. The present participants had only migraine headache and no other health conditions, and possible confounding factors that could have affected hs-CRP levels and WMHs were avoided such as vascular risk factors, inflammatory disease, or chronic illness. We did not include patients who had cerebral ischemic infarcts. Therefore, it is likely that we studied earlier stages of WMHs in migraine patients. The absence of an association between hs-CRP levels and WMHs may support the hypothesis that the hs-CRP level does not affect the brain pattern or severity of WMHs that are associated with migraine disease. The WMHs on MRI in migraine patients may be associated with migraine disease and may be determined genetically [18, 40].

In the present study, the anatomic location of WMHs was different between patients who had different migraine subtypes and control subjects. Only migraine patients with aura had periventricular WMHs. The WMHs were located mostly in the subcortical region and frontal lobe (Table 2) The WMHs of the control subjects were located only in the subcortical region. The distribution of subcortical and juxtacortical WMHs was not different between migraine patients with or without aura (Table 2). A previous cross-sectional population study also showed a higher association between deep WMHs and migraine in patients who had migraine with than without aura [20]. In contrast with our study, there were no differences in the location of periventricular WMHs between migraine patients and control subjects. It was proposed that different mechanisms may affect the development of deep and periventricular WMHs in patients who have migraine with or without aura [18]. In addition, we did not find any differences between female and male migraine patients in the presence, number, or distribution of WMHs (Table 4). In contrast with the present study, the CAMERA 1 and 2 studies showed that women who had migraine, especially migraine without aura, had a higher incidence of deep WMHs and deep WMH progression than control subjects [18, 19]. In the previous studies, migraine patients were older and had vascular risk factors, and the WMH classification was different than in the present study.

In the present study, we observed no associations between WMHs and migraine headache characteristics, similar to previous studies [19]. In contrast with the present findings, previous studies reported a positive correlation between WMHs and migraine headache characteristics, but in the previous studies, the patients were older and had more comorbid conditions than the present study participants [14, 18, 20].

Although revisions in 2010 simplified the McDonald criteria, allowing an earlier diagnosis of multiple sclerosis (enabling establishment of dissemination in space (DIS) and dissemination in time (DIT) even with a single scan), they imposed an importance to the issue of excluding alternative diagnoses. With the increasing use of brain MRI, the finding of asymptomatic intracranial abnormalities has increased, and there is increased awareness of MRI findings suggestive of multiple sclerosis in patients without typical multiple sclerosis symptoms [41]. The importance of this problem was highlighted by the definition of the entity termed *radiologically-isolated syndrome* (RIS) [33]. Half of the patients who have RIS had their initial MRI because of headache, and characteristic imaging features and clinical associations of WMHs in migraine should be determined [21]. Therefore, many studies have suggested new and different definitions of periventricular and juxtacortical lesions because they were not precisely defined previously [42]. However, newer definitions may cause confusion instead of enabling an accurate classification of WMHs that are observed incidently on brain MRI scans. In our study, we also tried to differentiate incidental MRI lesions from imaging characteritics and factors associated with migraine; we observed that the prevelance of WMHs was 4.35-times higher in migraine patients than control subjects. Although our cohort included few patients, only 1 of 69 patients with migraine (1.4%) satisfied the revised 2010 McDonald criteria for multiple sclerosis [32], consistent with another study that reported that the 2010 McDonald criteria were satisfied in 4 of 44 patients with migraine (9%) [21, 32]. In another headache study, 2.4%-7.1% patients satisfied the Barkhof criteria and 24.4%-34.5% patients satisfied the McDonald criteria; in that study, the Barkhof and McDonald criteria were modified and included migraine patients with unknown medical history [42]. Therefore, these findings may have represented a false positive finding. Yet, it is important to be aware of WMHs because of the potential for diagnostic confusion. Aging, vascular risk factors, and inflammatory disease are associated with WMHs [21, 20]. Nevertheless, none of our patients with migraine satisfied the Barkhof criteria. Therefore we suggest caution in interpreting asymptomatic MRI findings, especially in migraine patients, to avoid overdiagnosis. The Barkhof criteria may be more sensitive than McDonald 2010 multiple sclerosis criteria. The size of WMHs may be useful in differentiating migraine from demyelinating disease.

## Conclusions

The present study showed that high levels of hs-CRP may be a marker of the proinflammatory state in migraine patients. However, the absence of correlation between hs-CRP and WMHs suggests that hs-CRP is not causally involved in the pathogenesis of WMHs in migraine patients. The WMHs were located mostly in the frontal lobe and subcortical area. The size and location of the WMHs may be important to differentiate WMHs from demyelination. Further prospective studies are justified to evaluate the relation between migraine and WMHs.

## Authors’ information

A. Yilmaz Avci, assistant professor; H. Lakadamyali, associate professor; S. Arikan, assistant professor, U.S. Benli, professor; M. Kilinc, associate professor.

## Abbreviations

CRP: C-reactive protein
WMHs: white matter hyperintensities
FLAIR: fluid attenuated inversion recovery
hs-CRP: high sensitivity C-reactive protein
MRI: magnetic resonance imaging.
